# A new chart of hydraulic fracture height prediction based on fluid–solid coupling equations and rock fracture mechanics

**DOI:** 10.1098/rsos.180600

**Published:** 2018-10-10

**Authors:** Xiaoqiang Liu, Zhanqing Qu, Tiankui Guo, Dongying Wang, Qizhong Tian, Wei Lv

**Affiliations:** 1 College of Petroleum Engineering, China University of Petroleum, Qingdao 266580, People's Republic of China; 2 Petroleum Engineering Technology Research Institute of Sinopec Shengli Oilfield, Dongying 257000, People's Republic of China

**Keywords:** hydraulic fracture height, fluid–solid coupling equations, rock fracture mechanics, ABAQUS extended finite-element, acoustic wave logging

## Abstract

The conventional method to predict hydraulic fracture height depends on linear elastic mechanics, and the typical Gulrajani–Nolte chart fails to reflect fracture height when the net pressure in the fracture is too high. Based on fluid–solid coupling equations and rock fracture mechanics, a new chart is obtained by the ABAQUS extended finite-element method. Compared with the Gulrajani–Nolte chart, this new chart shows that longitudinal propagation of hydraulic fracture is still finite when the net pressure in the fracture is higher than *in situ* stress difference between reservoir and restraining barrier. The barrier has a significant shielding effect on the longitudinal propagation of hydraulic fracture, and there is a threshold for an injection rate of fracturing fluid to ensure hydraulic fracture propagates in the barrier. Fracture height decreases with the increase of *in situ* stress difference. When the ratio of net pressure to *in situ* stress difference is less than 0.56, the propagation of hydraulic fracture is completely restricted in the reservoir. Hydraulic fracturing parameters in Well Shen52 and Well Shen55 are optimized by using the new chart. Array acoustic wave logging shows that the actual fracture height is at an average error within 14.3% of the theoretical value, which proves the accuracy of the new chart for field application.

## 1. Introduction

Tight sandstone gas reservoir is an important type of unconventional reservoir. The successful development of tight sandstone gas reservoirs has a great significance for ensuring the stability of gas production in China. Shenmu block is located in the eastern Ordos Basin of China and has been proved to be rich in natural gas. Taiyuan formation is the major layer of this block with low permeability (ranging from 0.1 to 0.5 mD) and low porosity (ranging from 5% to 9%). Large-scale hydraulic fracturing is the key technology for developing tight sandstone gas reservoir. The site hydraulic fracturing operation shows that aquifer and gas reservoir are separated by a restraining barrier in Taiyuan formation, and there is a great difference in physical property between reservoir rock and barrier rock. Improper hydraulic fracturing operation can easily cause fractures to penetrate the barrier, and water in aquifers will flow into the production well through fractures. Thus, it is necessary to predict hydraulic fracture height before hydraulic fracturing operation is conducted.

**Figure 1.**
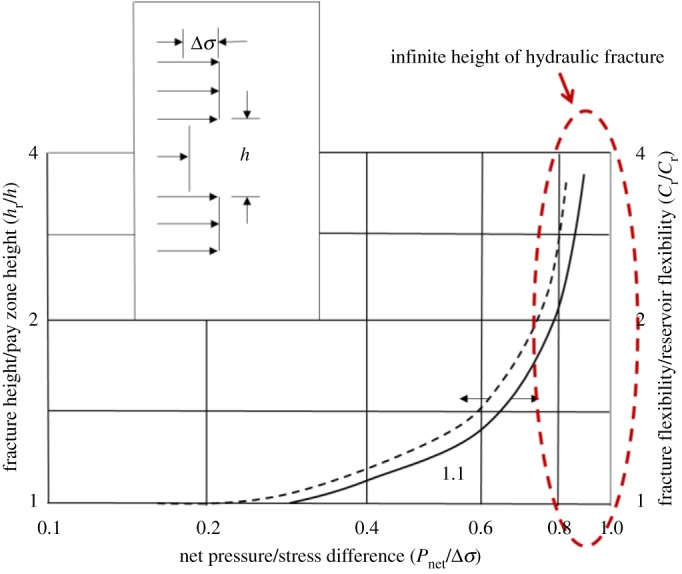
The Gulrajani–Nolte chart.

The longitudinal propagation of hydraulic fracture is influenced by rock properties and operation parameters. The early study mainly depends on linear elastic mechanics and many scholars have carried out related research. Biot *et al.* [1] combined theoretical and experimental investigation of fracture penetration through an interface. It is commonly inferred that fractures are not able to propagate across a bedding plane if it is weak and has a high tendency to slip. Warpinski & Teufel [2] presented that *in situ* stress difference was the primary factor affecting the longitudinal propagation of hydraulic fracture. Teufel & Clark [3] put forward that the weak interfacial shear strength of barrier rock and the increase of the minimum horizontal compressive stress could inhibit the vertical growth of hydraulic fractures in the layered rock. Daneshy [4] considered that shear slip was beneficial to restraining longitudinal propagation of hydraulic fractures. Nolte & Smith [5] established the double logarithmic relationship between the net pressure in fracture and geometric size of the hydraulic fracture. Wei *et al.* [6] and Liu *et al.* [7] analysed the fracture initiation and propagation mechanism in tight sandstone by using true tri-axial hydraulic fracturing experiments. Rock properties, natural fractures and injection rates of fracturing fluid were three major factors affecting fracture longitudinal propagation. Guo *et al.* studied the effects of natural fracture on hydraulic fracture propagation by numerical simulation and physical experiments, and proposed that hydraulic fracture will cross, be arrested and restart under different conditions [8–11]. Simonson *et al.* [12] analysed the elastic property of a rock, *in situ* stress and the pressure gradient on fracture containment according to linear elastic fracture mechanics. Based on the theory of linear elasticity, the Gulrajani–Nolte chart (shown in figure 1) was presented that used net pressure and *in situ* stress difference to predict fracture height [13]. The Gulrajani–Nolte chart showed that only when the net pressure reached a critical value that hydraulic fracture could penetrate the interface. This chart also showed the relation curve of net pressure and fracture height when the net pressure was less than *in situ* stress difference between reservoir and restraining barrier. However, the Gulrajani–Nolte chart is invalid when net pressure is higher than *in situ* stress difference. As is shown in the Gulrajani–Nolte chart, when net pressure is closed to *in situ* stress difference, fracture height tends to be infinite, which is not in conformity with the fact.

As a novel technique, the ABAQUS software has been proved to be an effective method to simulate hydraulic fracturing. It can take into account the dynamic changing process of reservoir rock porosity, permeability, pore pressure and fracture surface filtration. Many scholars have carried out a related application in recent years. Qu *et al.* [14–16] introduced the principle of the ABAQUS extended finite-element method to simulate fracture propagation, and the accuracy of numerical simulation results was verified by physical experiments. Haddad *et al.* [17,18] proposed an XFEM-based CZM model in ABAQUS to simulate the fracture initiation and propagation. This numerical technique was validated by comparing the solution with KGD solution. Dehghan *et al.* [19,20] analysed the propagation of hydraulic fractures at its intersection with natural fractures by ABAQUS. Experimental models showed a good agreement with numerical results, which proved ABAQUS a valid technique to simulate fracture propagating at interactions. Li *et al.* [21] studied the effect of reservoir rock, barrier and interfacial properties on hydraulic fracture height control by cohesive element based on the ABAQUS. Rock elastic modulus, *in situ* stress and tensile strength are considered to have an important effect on fracture height. But there is no quantitative conclusion or chart for field operation.

At present, the prediction of hydraulic fracture height in oilfields mainly depends on operation experience, or using the Gulrajani–Nolte chart for reference, and the study method mainly depends on linear elastic mechanics. However, the limitation of the Gulrajani–Nolte chart has restricted its wide application. In view of the above problem, a numerical simulation model of longitudinal propagation of hydraulic fracture in tight sandstone reservoir is established by the ABAQUS extended finite-element method. It reveals the influence of operation parameters and rock properties on fracture longitudinal propagation. A new relationship curve between net pressure and *in situ* stress difference is obtained. This new chart makes up the deficiency of the Gulrajani–Nolte chart. A hydraulic fracturing operation in Shenmu block is designed using the new chart.

## 2. Establishment of hydraulic fracturing model

The hydraulic fracturing model consists of three parts: (i) fluid–solid coupling—fracturing fluid and fluid in formation pores have an influence on rock matrix stress, porosity and permeability; (ii) fracture initiation—the hydraulic pressure reaches the tensile strength of the rock, causing the rock to begin to rupture; (iii) fracture propagation—after hydraulic fracture is opened, it extends forward with the continuous injection of fracturing fluid. Numerous researches have shown that the matrix poroelastic deformation (also known as ‘back-stress’ in hydraulic fracturing literature) can affect the net pressure, fracture length, leak-off, aperture and the interactions between hydraulic fractures [22,23]. In this paper, we proposed a stress equilibrium equation of rock, fluid continuity equation of liquid flowing in a porous medium and fracture flow model. The stress equilibrium equation of rock is coupled to the fluid flow continuity equation in the porous medium through the effective stress concept. The fluid flow continuity equation in a porous medium is linked to the fracture flow model by a leak-off coefficient. Fracturing fluid and fluid in formation pores have an influence on rock matrix stress, porosity and permeability. And matrix poroelastic deformation also affects the fracturing flow in turn.

### 2.1. Fluid–solid coupling equation and discretization of finite-element

#### 2.1.1. Stress equilibrium equation of rock

The effective stress for rock matrix saturated with single-phase fluid is defined as [24]

2.1 $$\sigma^{\prime }=\sigma +\alpha p_{m}I,$$

where $\sigma^{\prime }$ is the effective stress, Pa; *σ* is the total stress, Pa; *α* is the Boit coefficient; *p*_m_ is the matrix fluid pressure, Pa; ***I*** is the second-order identify tensor. The Boit coefficient is defined as

2.2 $$\alpha =1-\frac{K}{K_{s}},$$

where *K* and *K_s_* are the bulk moduli of the porous rock and of the rock matrix material, respectively, Pa.

The stress and strain relationship can be expressed in incremental form as

2.3 $$d\sigma^{\prime }=D_{\mathrm{ep}}(d\varepsilon -d\varepsilon_{l}),$$

where $D_{\mathrm{ep}}$ is the elastic–plastic matrix; $d\varepsilon_{l}$ is the rock compression strain caused by pore fluid pressure, which can be written as

2.4 $$d\varepsilon_{l}=-m\frac{dp_{o}}{3K_{S}},$$

where *p_o_* is the rock pore pressure, Pa; *m* = [1, 1, 1, 0, 0, 0].

The stress equilibrium equation of rock can be expressed by the virtual work principle. The virtual work of rock is equivalent to the virtual work generated by the forces (including body force and surface force) acting on the rock:

2.5 $$\int_{V}\delta \varepsilon^{T}d\bar{\sigma }\;dV-\int_{V}\delta u^{T}df\;dV-\int_{S}\delta u^{T}d\tau \;dS=0,$$

where δε is the virtual displacement, m; δu is the virtual strain; τ is the surface force, N m^−2^; *f* is the volume force, N m^−3^; dS is the area element, m^2^; dV is the volume element, m^3^.

Combined formula (2.1)–(2.4), the formula (2.5) can be written as

2.6 $$\int_{V}\delta \varepsilon^{T}D_{\mathrm{ep}}\left(d\varepsilon +\alpha m\frac{dp_{o}}{3K_{S}}\right)dV-\int_{V}\delta \varepsilon^{T}\alpha mdp_{o}\;dV-\int_{V}\delta u^{T}df\;dV-\int_{S}\delta u^{T}d\tau \;dS=0.$$

After some manipulation, the stress equilibrium equation of rock skeleton deformation during the hydraulic fracturing process is written as [25,26]

2.7 $$\begin{array}{rl} & \int_{V}\delta \varepsilon^{T}D_{\mathrm{ep}}\frac{d\varepsilon }{dt}\;dV+\int_{V}\delta \varepsilon^{T}D_{\mathrm{ep}}\left[\alpha m\frac{\left(s_{o}+p_{o}\varepsilon \right)}{3K_{s}}\frac{dp_{o}}{dt}\right]dV\\ & \;-\int_{V}\delta \varepsilon^{T}\alpha m(s_{o}+p_{o}\xi )\frac{dp_{o}}{dt}\;dV=\int_{S}\delta u^{T}\frac{d\tau }{dt}\;dS+\int_{V}\delta u^{T}\frac{df}{dt}\;dV \end{array}$$

where $D_{\mathrm{ep}}$ is the elastic–plastic matrix; d*t* is the time step, s; $S_{o}$ is the saturation; $p_{o}$ is the rock pore pressure, Pa; $\xi =ds_{o}/dp_{o}$.

#### 2.1.2. Fluid continuity equation of liquid flowing in a porous medium

The flow of liquid in porous media is Darcy flow. Darcy's Law for the porous medium flow may be written as

2.8 $$v_{w}=-\frac{1}{n_{w}g\rho_{w}}k\cdot \left(\frac{\partial p_{w}}{\partial x}-\rho_{w}g\right),$$

where *v_w_* is the liquid flow velocity, m s^−1^; *k* is the permeability, ×10^–15^ µm^2^; *n_w_* is the ratio of liquid volume to total volume of rock; *p_w_* is the pressure difference, Pa; $\rho_{w}$ is the liquid density, kg m^−3^.

According to the principle of mass conservation, for a certain volume of rock, the total mass of fluid flowing into the rock within a certain time is equal to the mass of increment and outflow of the rock. The law of mass conservation for fluid flowing in porous media can be written as follows [25]:

2.9 $$\begin{array}{rl} & s_{W}\alpha \left(m^{T}-\frac{m^{T}D_{\mathrm{ep}}}{3K_{S}}\right)\frac{d\varepsilon }{dt}-\nabla^{T}\left[k(\frac{\nabla p_{W}}{\rho_{W}}-g)\right]+\left\{\xi n+n_{w}\frac{s_{W}}{K_{W}}+s_{W}\left[\frac{1-n_{w}}{3K_{S}}-\frac{m^{T}D_{\mathrm{ep}}\alpha m}{{\left(3K_{s}\right)}^{2}}\right](s_{W}+p_{W}\xi )\right\}\frac{dp_{W}}{dt}=0, \end{array}$$

where $s_{W}$ is the saturation of fluid in porous rock; $K_{W}$ is the fluid volume modulus, GPa.

#### 2.1.3. Fracture flow model

**Figure 2.**
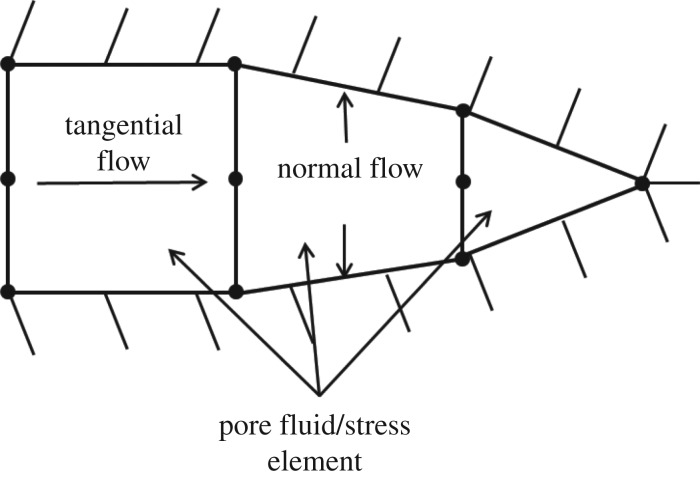
Fluid flow pattern in pore fluid/stress element.

The flow of fluid in fracture includes tangential flow and normal flow, as shown in figure 2. Tangential flow mode is used to describe the fracturing fluid flow in the fracture, and normal flow model is used to simulate the fracturing fluid leak-off into rock matrix. The fluid is hypothesized to be incompressible Newtonian fluid, so the tangential flow is governed by the lubrication equation:

2.10 $$qd=-k_{t}\nabla p,$$

where *q* is the tangential flow rate of the fluid, m s^−1^; *k_t_* is the tangential permeability; ∇p is the tangential flow fluid pressure gradient, Pa m^−1^; *d* is the fracture aperture, *m*. The tangential permeability is defined according to Reynold's equation:

2.11 $$k_{t}=\frac{d^{3}}{12\mu },$$

where μ is the viscosity of fracture fluid, Pa s.

**Figure 3.**
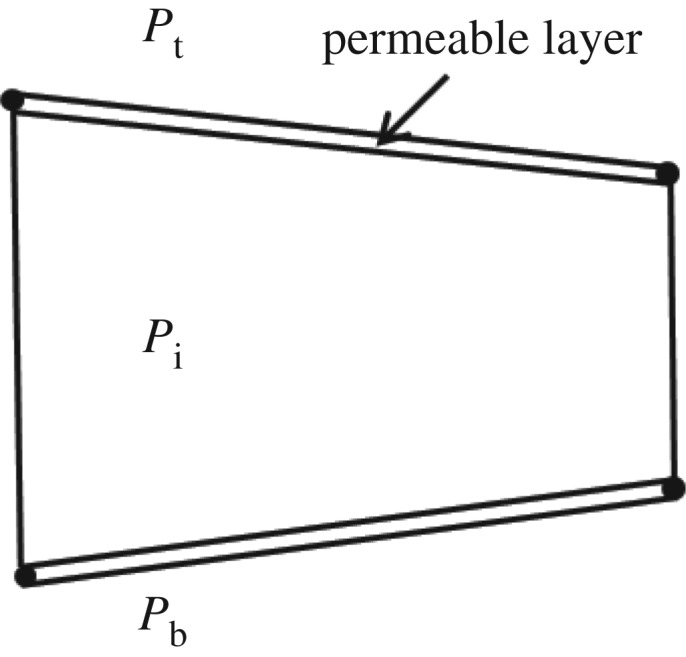
Leak-off coefficient illustrated as permeable layer.

The normal flow represents the fracturing fluid leak into the porous medium and is modelled by defining a leak-off coefficient for the porous rock. This coefficient can be interpreted as the permeability of the layers on either side of the surface, shown in figure 3. The normal flow equation can be written as [8]

2.12 $$\left.\begin{array}{c} \;q_{t}=C_{t}(P_{i}-P_{f})\\ \;\mathrm{and}\;q_{b}=\;C_{b}(P_{i}-P_{b}), \end{array}\right\}$$

where $q_{t}$ and $q_{b}$ are the flow rates into the top and bottom surfaces, respectively, m s^−1^; $c_{t}$ and $c_{b}$ are leak-off coefficients of the top and bottom surfaces, respectively; $p_{t}$ and $p_{b}$ are the pore pressure on the top and bottom surfaces, respectively, Pa; $p_{i}$ is the fluid pressure in the fracture, Pa.

#### 2.1.4. Dynamic change equation of rock porosity and permeability

During hydraulic fracturing, the porosity and permeability of rock will change with hydraulic fracture propagation. The dynamic change equation of rock porosity and permeability can be described as follows [27]:

2.13 $$\varphi =\frac{\varphi_{0}-\varepsilon_{v}}{1-\varepsilon_{v}}$$

and

2.14 $$k=k_{0}\frac{1}{1-\varepsilon_{v}}{\left(1-\frac{\varepsilon_{v}}{\varphi_{0}}\right)}^{3},$$

where φ is the dynamic porosity of rock; $\varphi_{0}$ is an initial porosity of rock; $\varepsilon_{v}$ is the rock volume strain; *k* is the dynamic permeability of rock, μm^2^; $k_{0}$ is an initial permeability of rock, μm^2^.

#### 2.1.5. ABAQUS discretization of finite-element and fluid–solid coupling equation

By introducing the shape function, the transient variation of each parameter can be discretized into the fluid–solid coupling equation about *in situ* stress, strain, porosity and permeability of the rock. The final fluid–solid coupling equation can be written as [28]

2.15 $$\left[\begin{array}{cc} K & C\\ E & G \end{array}\right]\frac{d}{dt}\left\{\begin{array}{c} \bar{u}\\ \bar{\;p_{o}} \end{array}\right\}+\left[\begin{array}{c} 00\\ 0F \end{array}\right]\;\left\{\begin{array}{c} \bar{u}\\ \bar{\;p_{o}} \end{array}\right\}=\left\{\begin{array}{c} \frac{df}{dt}\\ \hat{f} \end{array}\right\}.$$

In which

2.16 $$\left.\begin{array}{rl} K= & \int_{V}B^{T}D_{\mathrm{ep}}BdV,\\ C= & \int_{V}B^{T}D_{\mathrm{ep}}\alpha m\frac{\left(s_{o}+\xi p_{o}\right)}{3K_{s}}N_{p}\;dV-\int_{V}B^{T}(s_{o}+\xi p_{o})\alpha mN_{p}\;dV,\\ E= & \int_{V}N_{P}^{T}\left[\alpha s_{o}\left(m^{T}-\frac{m^{T}D_{\mathrm{ep}}}{3K_{s}}\right)B\right]\;dV,\\ F= & \int_{V}{\left(\nabla N_{p}\right)}^{T}kk_{T}\nabla N_{p}\;dV,\\ G= & \int_{V}N_{P}^{T}\left\{s_{o}\left[\left(\frac{1-n}{K_{s}}-\frac{m^{T}D_{\mathrm{ep}}\alpha m}{{\left(3K_{s}\right)}^{2}}\right)\right]\cdot (s_{o}+p_{o}\xi )+\xi n+n\frac{s_{o}}{K_{o}}\right\}N_{p}\;dV,\\ df= & \int_{V}N_{u}^{T}df\;\;dV+\int_{S}N_{u}^{T}d\tau \;dS\\ \;\mathrm{and}\;\overset{⌢}{f}= & \int_{S}N_{p}^{T}q_{\mathrm{ob}}\;dS-\int_{V}{\left(\nabla N_{p}\right)}^{T}kk_{r}g\;dv, \end{array}\right\}$$

where *B* and *N_u_* are the vector matrix of shape function; $\bar{u}$ is displacement of an element node, m; $\bar{\;p_{0}}$ is pore pressure of element node, Pa; *N_p_* is shape function; *n* is the unit normal direction of the boundary; $q_{\mathrm{ob}}$ is the fluid rate at the boundary.

### 2.2. Criteria for fracture initiation

Based on the previous research, the maximum principal stress criterion shows reliable results as the criterion of fracture initiation [29]. The basic principle is that rock breaks and damage forms when hydraulic pressure is greater than the critical maximum principal stress of the rock.

The maximum principal stress criterion is described as

2.17 $$f=\left\{\frac{\left(\sigma_{\max}\right)}{\sigma_{\max}^{a}}\right\},$$

where $\sigma_{\max}^{a}$ is the critical maximum principal stress of rock; $\sigma_{\max}$ is the maximum stress of rock.

### 2.3. Damage evolution law

Damage evolution process is defined as the energy required for further destruction of rock after initial damage. The Benzeggagh–Kenane criteria are introduced to hydraulic fracture growth when fracture initiation occurs and expressed as [30]

2.18 $$G_{n}^{C}+(G_{s}^{C}-G_{n}^{C}){\left\{\frac{G_{s}}{G_{T}}\right\}}^{\eta }=G^{C},$$

where $G_{s}$ and $G_{T}$ are the fracture energy release rate at X and Y directions, respectively; η is a constant related to the material property; $G^{C}$ is the critical fracture energy release rate of composite fracture; $G_{s}^{C}$ and $G_{n}^{C}$ are the critical energy release rate at tangential and normal direction, respectively.

**Figure 4.**
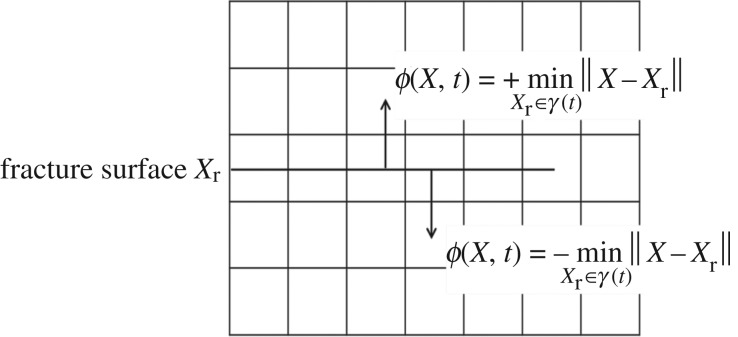
Schematic diagram of fracture propagation and ϕ function.

The horizontal set method can simulate the motion of the fracture interface without any remeshing. The ϕ function is updated by calculating the zero level set of ψ and $\phi_{i}$ to realize the simulation of fracture propagation [31]. The updating and evolution of the ϕ function is actually a progress of simulating fracture propagation (shown in figure 4). The symbol distance function in the horizontal set method can be written as

2.19 $$\phi (X,t)=\pm \underset{X_{r}\in \gamma (t)}{\min}∥X-X_{r}∥,$$

where ϕ(X,t) is used to describe the fracture surface. When *X* is located above the fracture defined by γ(t), the value of the formula is positive, and vice versa.

### 2.4. Numerical model

**Figure 5.**
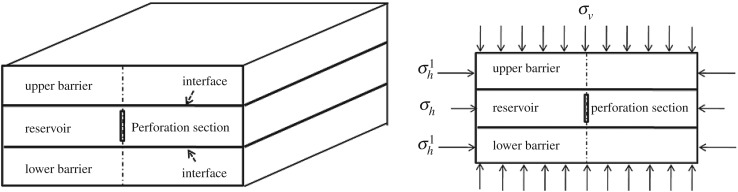
Schematic diagram of a three-dimensional model of barrier-reservoir-barrier.

Based on geological data of a tight sandstone reservoir in Shenmu block of Changqing Oilfield of China, a three-dimensional numerical model of barrier–reservoir–barrier is established (shown in figure 5). The size of this model is 50 × 50 × 9 m with the same thickness of reservoir and a barrier of 3 m separately. Perforation direction is along the maximum principal stress direction. Basic parameters are shown in table 1.

**Table 1.** Basic parameters of numerical simulation model.

parameters	value	parameters	value
horizontal maximum principal stress of reservoir	40 MPa	horizontal minimal principal stress of reservoir	32 MPa
horizontal maximum principal stress of restraining barrier	52 MPa	horizontal minimal principal stress of restraining barrier	44 MPa
Young's modulus of reservoir	36.8 GPa	Young's modulus of restraining barrier	24 GPa
Poisson ratio of reservoir	0.24	Poisson ratio of restraining barrier	0.34
initial porosity of reservoir	7%	initial porosity of restraining barrier	2%
reservoir permeability	0.113 × 10^–15^ µm^2^	restraining barrier permeability	0.02 × 10^–15^ µm^2^
tensile strength of reservoir	6 MP	tensile strength of restraining barrier	8 MP
normal fracture energy release rate in reservoir	28 N mm^−1^	shear fracture energy release rate in barrier	38 N mm^−1^
normal fracture energy release rate in reservoir	28 N mm^−1^	shear fracture energy release rate in barrier	38 N mm^−1^
Biot coefficient	0.179	leak-off coefficient	3.4 × 10^–7^ m s^−0.5^
overburden stress	62 MPa	initial pore pressure	22 MPa
injection rate of fracturing fluid	6.5 m^3^ min^−1^	fracturing fluid viscosity	50 MPa s

## 3. Analysis of simulation results

In the process of hydraulic fracturing, the induced stress resulting from a hydraulic fracture is spread to the restraining barrier ahead of fracture height. The existence of a restraining barrier will influence the value of stress at the tip of fracture and the distribution of stress field. As a result, hydraulic fracture propagation in reservoir and barrier is different from that in single lithology. To study the longitudinal propagation of hydraulic fracture, it is necessary to analyze the influence of the interface on the fracture tip stress field at first.

**Figure 6.**
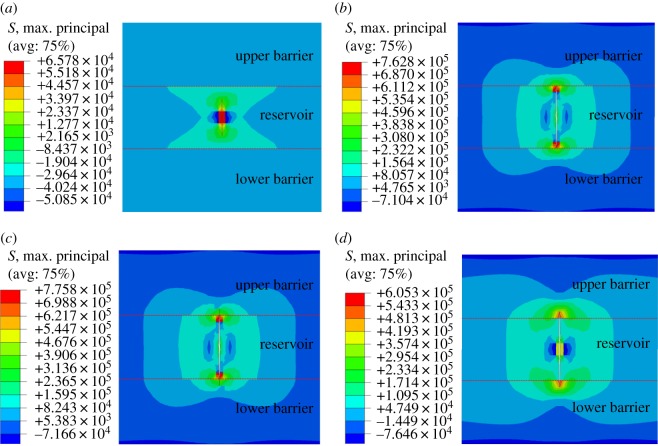
Longitudinal propagation of fracture and stress field distribution under different injection time of fracturing fluid: (*a*) 100 s, (*b*) 300 s, (*c*) 500 s, (*d*) 700 s.

Figure 6 shows the longitudinal propagation of hydraulic fracture in the reservoir and restraining barrier under the same time step (200 s). At the initial stage of hydraulic fracturing, stress concentration occurs at the fracture tip. When the maximum tensile stress at fracture tip is greater than the tensile strength of reservoir rock, the reservoir will be cracked and hydraulic fracture will stretch upward with a continuous injection of fracturing fluid. Before hydraulic fracture propagates to the interface, the restraining barrier has a certain inhibitory effect on the longitudinal propagation of induced stress (figure 6*a*). After hydraulic fracture reaches the interface, the stress at fracture tip continues to grow. Before the maximum principal stress reaches the tensile strength of the restraining barrier rock, the longitudinal propagation of hydraulic fracture is restricted, manifested as width increase and unchanged height (figure 6*b*). When hydraulic fracture penetrates the interface, hydraulic fracture propagation is significantly affected by rock property of restraining barrier. The propagation speed of hydraulic fracture in barrier zone is obviously lower than that in the reservoir (figure 6*c*). The existence of a restraining barrier has an obvious blocking effect on the longitudinal propagation of hydraulic fracture, leading to a slow increase of fracture height and a further increase of fracture width (figure 6*d*).

### 3.1. Injection rate of fracturing fluid

**Figure 7.**
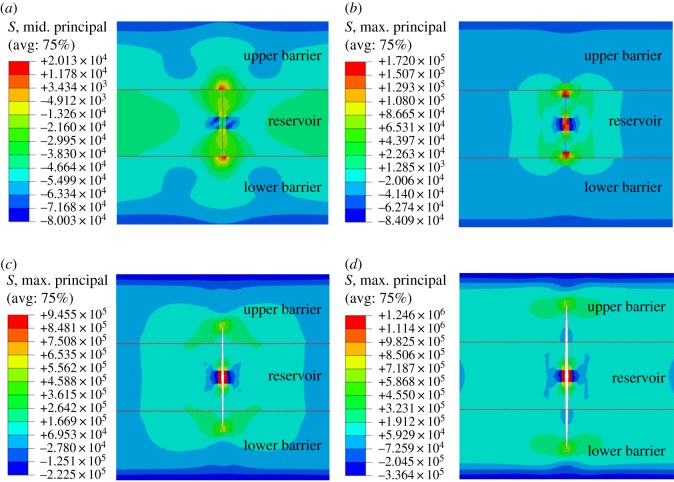
Effect of injection rate of fracturing fluid on longitudinal propagation of hydraulic fracture. (*a*) 2 m^3^ min^−1^, (*b*) 4 m^3^ min^−1^, (*c*) 6 m^3^ min^−1^, (*d*) 8 m^3^ min^−1^.

The effect of injection rate of fracturing fluid (2, 4, 6 and 8 m^3^ min^−1^) on the longitudinal propagation of hydraulic fracture is analysed, and simulation results are shown in figure 7.

There is a threshold for an injection rate of fracturing fluid to realize hydraulic fracture penetrating interface and propagating in the barrier. Figure 7 shows that when injection rate is lower than 4 m^3^ min^−1^, hydraulic fracture propagation is strictly restricted in the reservoir. When injection rate increases to 6 m^3^ min^−1^, hydraulic pressure rises rapidly to form a high pressure in a short time, which is greater than the tensile strength of barrier rock, resulting in hydraulic fracture penetrating the interface and propagating in barrier.

### 3.2. Viscosity of fracturing fluid

**Figure 8.**
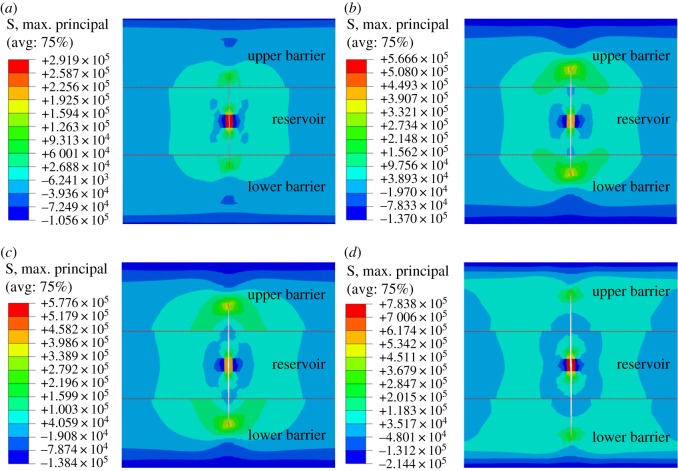
Effect of viscosity of fracturing fluid on longitudinal propagation of hydraulic fracture. (*a*) 5 mPa s, (*b*) 50 mPa s, (*c*) 100 mPa s, (*d*) 200 mPa s.

**Figure 9.**
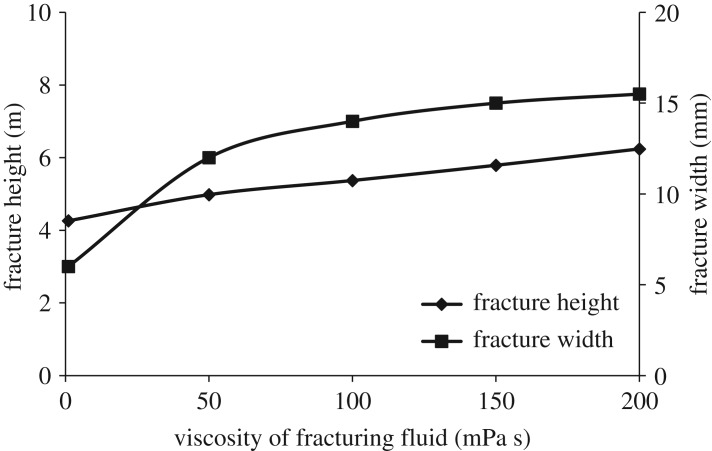
Variation curve of fracture height and fracture width with viscosity of fracturing fluid.

Tight sandstone is characterized by low permeability, small pores and high content of fillings. In order to protect the reservoir from damage, a fracturing fluid system with small molecular weight and low viscosity is often used during hydraulic fracturing in a tight sandstone reservoir. The longitudinal propagation of hydraulic fracture with a viscosity of fracturing fluid of 5, 50, 100, 150 and 200 mPa s is studied. Simulation results are shown in figures 8 and 9.

When slippery water (with a viscosity less than 10 mPa s) is used for fracturing, the longitudinal propagation of hydraulic fracture in barrier zone is weak, which is beneficial to controlling fracture height. But the fracture width is small, which is unfavourable to the movement of proppant. Fracture width increases noticeably when the viscosity of fracturing fluid exceeds 50 mPa s, which indicates that increasing the viscosity of fracturing fluid can improve fracture conductivity, but fracture height also increases with the increase of fracturing fluid viscosity. When the viscosity of fracturing fluid exceeds 100 mPa s, fracture width increases much slower but fracture height increases continuously, which indicates that the further increase of fracturing fluid viscosity will increase fracture propagation capacity in the barrier.

### 3.3. Injection time of fracturing fluid

**Figure 10.**
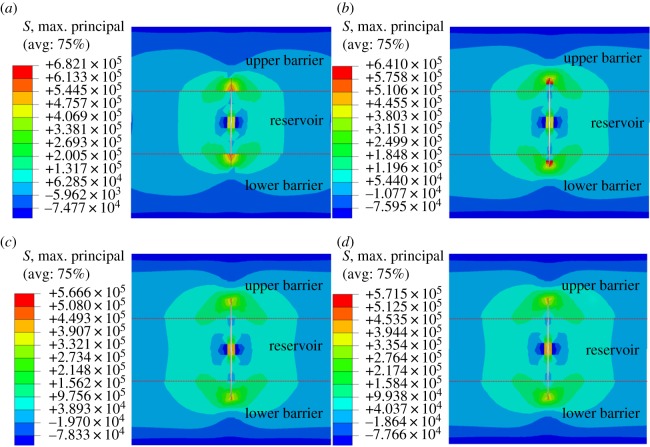
Influence of injection time of fracturing fluid on longitudinal fracture propagation. (*a*) 20 min, (*b*) 30 min, (*c*) 40 min, (*d*) 60 min.

**Figure 11.**
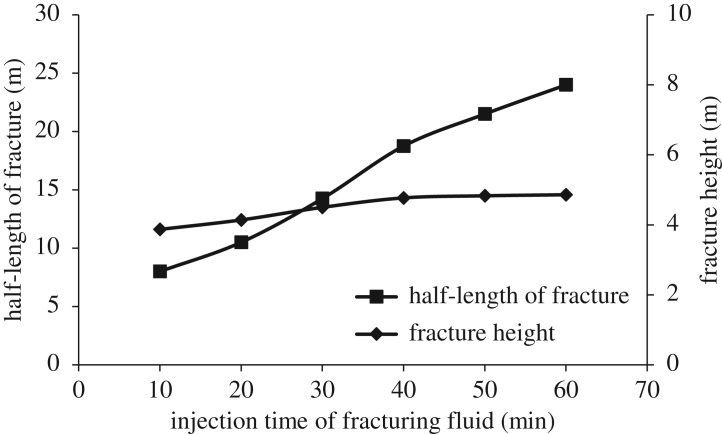
Variation curve of half-length of fracture and fracture height with time.

When fracture penetrates the interface and enters into a restraining barrier, further increase of fracture height requires the maximum tensile stress at fracture tip be greater than the tensile strength of barrier rock. Because the tensile strength of barrier rock is larger than that of reservoir rock, fracture propagation is limited in the longitudinal direction, and hydraulic fracture is more likely to propagate to the deep strata in the reservoir. As can be seen from figures 10 and 11, after fracturing fluid is injected for 40 min, fracture length increases with the continuous injection of fracturing fluid, while fracture height remains unchanged.

**Figure 12.**
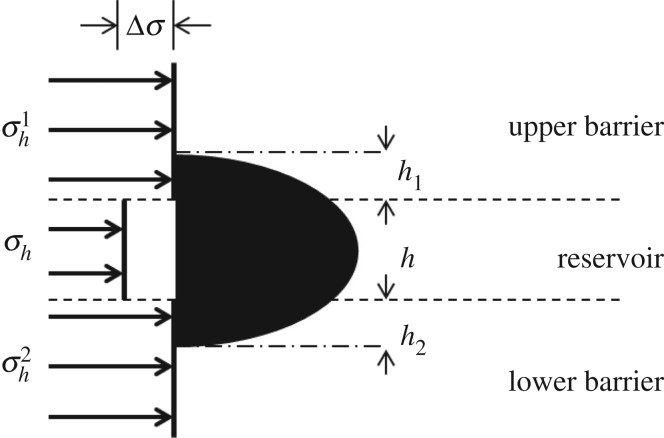
Schematic diagram of the critical thickness ratio between restraining barrier and reservoir.

The critical thickness ratio between the reservoir and the restraining barrier is defined as the ratio of the maximum thickness of hydraulic fracture in the barrier to the reservoir thickness. As shown in figure 12, when the stress difference between restraining barrier and reservoir is *Δσ*, the critical thickness ratio between upper barrier and reservoir is *h*_1_/*h*.

The critical thickness ratio can be used to predict whether hydraulic fracture completely penetrates the restraining barrier longitudinally. If the thickness of reservoir multiplied by the critical thickness ratio is greater than reservoir thickness, the hydraulic fracture can penetrate the barrier, otherwise, the hydraulic fracture will be restricted in the restraining barrier.

### 3.4. Leak-off of fracturing fluid

In the process of hydraulic fracturing, fracturing fluid can leak off into the rock matrix through fracture surfaces. Relevant research shows that larger leak-off will affect the fracture dimension [32]. According to the classical leak-off theory, the leak-off of fracturing fluid is controlled by the viscosity of fracturing fluid, the compressibility of fracturing fluid and the wall building property of fracturing fluid. And the leak-off coefficient in the reservoir region can be expressed as [33]

3.1 $$c=\sqrt{\frac{kC_{f}\phi }{\pi \mu_{f}}}\Delta P,$$

where *c* is the leak-off coefficient, m s^−0.5^; *k* is the reservoir permeability, m^2^; *C*_f_ is the fluid compression coefficient, 1/Pa; $\mu_{f}$ is the viscosity of fracturing fluid, Pa s; ΔP is the pressure difference between a hydraulic fracture and rock matrix, Pa.

**Figure 13.**
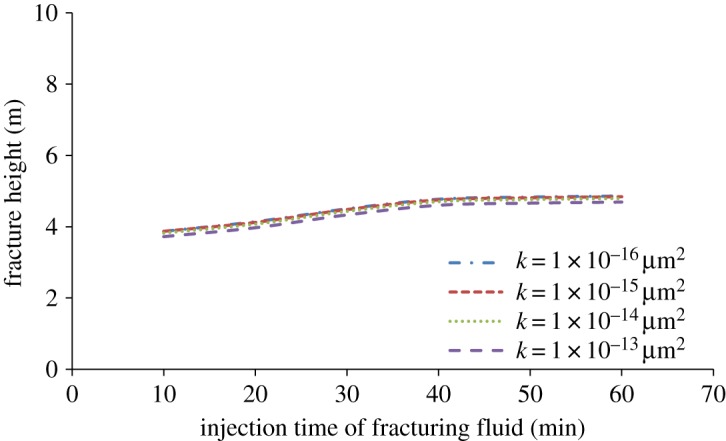
The influence of leak-off of fracturing fluid on fracture height with injection time.

Based on the formula (3.1) and data of table 1, when the permeability of rock matrix is 1 × 10^–16^, 1 × 10^–15^, 1 × 10^–14^ and 1 × 10^–13^ µm^2^, the leak-off coefficient of fracturing fluid can be calculated as 3.43 × 10^–7^, 8.92 × 10^–7^ , 3.56 × 10^–6^ and 1.13 × 10^–5^ m s^−0.5^, respectively. The longitudinal propagation of hydraulic fracture with different leak-off is studied. The simulation result is shown in figure 13.

The leak-off of fracturing fluid is very small in the range of permeability of 1 × 10^–16^ to 1 × 10^–13^ µm^2^. And the leak-off has little effect on fracture height when rock matrix permeability is smaller than 1 × 10^–14^ µm^2^, which can be proved in Salimzadeh & Khalili [32] simulation results as well. The permeability of tight sandstone gas reservoir is usually lower than 0.3 × 10^–15^ µm^2^ in Shenmu block of Changqing Oilfield, and the change of leak-off has little effect on fracture height in that range of permeability.

### 3.5. *In situ* stress difference between restraining barrier and reservoir

**Figure 14.**
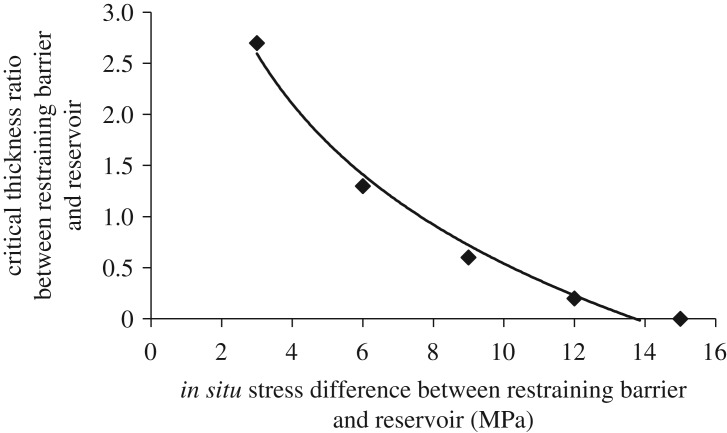
Relation curve of *in situ* stress difference and critical thickness ratio.

The change of *in situ* stress difference between restraining barrier and reservoir is bound to affect the longitudinal propagation of hydraulic fracture. Critical thickness ratio corresponding to the stress differences of 3, 6, 9, 12 and 15 MPa, respectively, are shown in figure 14.

The critical thickness ratio decreases with the increase of *in situ* stress difference. A large critical thickness ratio under a lower *in situ* stress difference indicates that the barrier has a weak blocking effect on the longitudinal propagation of hydraulic fracture. When the *in situ* stress difference is 3 MPa, the corresponding critical thickness ratio is 2.7. With the gradual increase of *in situ* stress difference, the critical thickness ratio decreases significantly. When *in situ* stress difference increases to 13.7, the fracture propagation will be completely restricted in the reservoir and the corresponding critical thickness ratio is zero.

### 3.6. Net pressure in fracture

The influence of injection rate of fracturing fluid, fracturing fluid viscosity and other operation parameters on fracture propagation is represented by the net pressure in a hydraulic fracture. In tight sandstone reservoirs, hydraulic fracture volume is equal to that of injected fracturing fluid when fracturing fluid leak-off is ignored. When the fracture height is *h*, the distribution of pressure in the hydraulic fracture can be expressed as [34]

3.2 $$\left.\begin{array}{rl} P_{\mathrm{net}}= & \frac{8\mu q\sqrt{L_{f}}}{3\pi hB^{2}}\left[\frac{3\pi \;\arcsin(x/L_{f})}{\sqrt{L_{f}^{2}-x^{2}}}+\frac{1.45}{L_{f}}\right]\\ & \;+\frac{\rho q\sqrt{L_{f}}}{54\pi hBt}\left[\frac{12x(2\arcsin(x/L_{f})-\pi )}{\sqrt{L_{f}^{2}-x^{2}}}-\frac{64}{3}+\frac{20x^{2}}{L_{f}^{2}}-9L_{f}^{2}\frac{\pi^{2}+4(\arcsin{\left(x/L_{f}\right)}^{2}-4\pi \arcsin(x/L_{f})}{L_{f}^{2}-x^{2}}\right]\\ & \;+\frac{C(t)}{\sqrt{L_{f}^{2}-x^{2}}}, \end{array}\right\}$$

where μ represents the viscosity coefficient of fracturing fluid; *q* represents the injection rate of fracturing fluid, m^3^ s^−1^; *ρ* represents the fracturing fluid density, kg m^−3^; *L*_f_ represents half-length of fracture, m; *h* represents fracture height, m; *E* represents Young's modulus of rock, GPa; $K_{\mathrm{Ic}}$ represents fracture toughness of rock; *x* represents any point along the lengthwise direction of hydraulic fracture; *t* represents the injection time of fracturing fluid, s; $B=4K_{\mathrm{Ic}}/\sqrt{\pi }E$; $C(t)=4\pi \mu q\sqrt{L_{f}}/hB^{3}.$

The net pressure in fracture corresponding to different *in situ* stress difference is calculated as in table 2.

**Table 2.** Net pressure in fracture corresponding to different *in situ* stress difference.

*in situ* stress difference between restraining barrier and reservoir (MPa)	3	6	9	12	15
net pressure in fracture (MPa)	7.7160	7.6863	7.6671	7.6574	7.7905
net pressure/*in situ* stress difference	2.5720	1.2511	0.8519	0.6381	0.5194
critical thickness ratio	2.7	1.3	0.6	0.2	0

**Figure 15.**
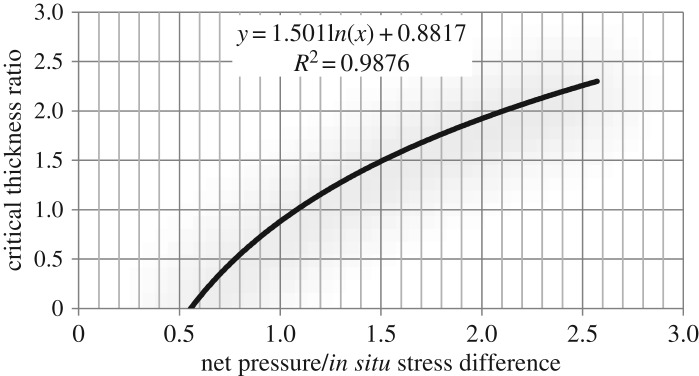
A new chart of hydraulic fracture height prediction.

The ratio of net pressure to *in situ* stress difference against the critical thickness ratio is curved and a new chart of hydraulic fracture height prediction as in figure 15.

The small ratio between net pressure and *in situ* stress difference indicates that hydraulic fracture propagation capacity in the longitudinal direction is weak. When the ratio is less than 0.56, hydraulic fracture propagation is completely confined to the reservoir and it cannot enter a restraining barrier longitudinally. Compared with the Gulrajani–Nolte chart, this new chart shows that longitudinal propagation of hydraulic fracture is still finite when the net pressure in fracture reaches or is higher than *in situ* stress difference between reservoir and restraining barrier. The new chart can be used to guide the establishment of operation parameters of hydraulic fracturing in the oilfield. If reservoir thickness and expected fracture height are given, the corresponding net pressure in the hydraulic fracture can be calculated according to the chart, and hydraulic fracturing operation parameters can be further determined.

## 4. Field application

Well Shen52 and Well Shen55 are located in Shenmu tight sandstone gas reservoir of Changqing oilfield of China. Operation parameters of hydraulic fracturing with controlling fracture height are optimized in those two wells by using the numerical simulation results. Fracture is the main cause of increased strata anisotropy, and array acoustic logging can be used to detect the anisotropy of strata to evaluate the longitudinal propagation of hydraulic fracture [35]. The significant increase of anisotropy detected by array acoustic logging before and after fracturing can be used to reflect the height of hydraulic fracture.

### 4.1. Well Shen52

**Figure 16.**
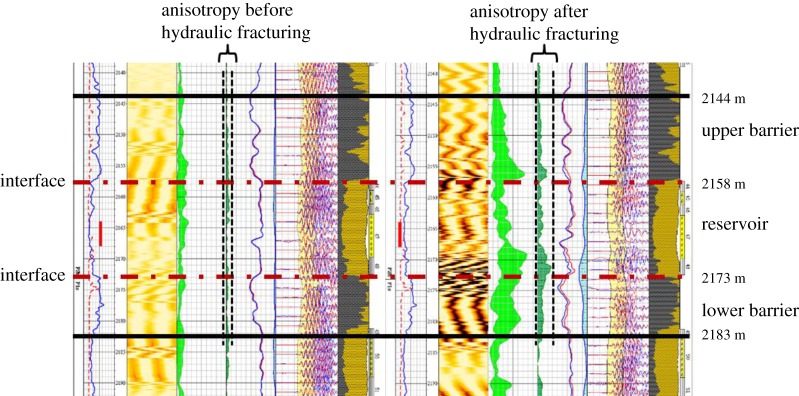
Interpretation results of array acoustic wave logging of Well Shen52.

The production layer is 2158–2173 m deep, with the maximum horizontal principal stress of 45 MPa and minimum horizontal principal stress of 33 MPa (shown in figure 16). The average porosity, permeability and gas saturation of the reservoir are 4.635%, 0.26 × 10^–15^ µm^2^ and 61.45%, respectively. The lithology of the upper barrier and the lower barrier is mudstone. The minimum horizontal principal stress difference between the upper barrier and reservoir is 7.9 MPa, and the minimum horizontal principal stress difference between the lower barrier and reservoir is 8.3 MPa. The lower barrier is 2173–2183 m deep (with a thickness of 10 m) and the thickness ratio between the lower barrier and reservoir is 0.67. According to the numerical result (figure 15), the corresponding net pressure in the hydraulic fracture is 7.19 MPa. Injection rate of fracturing fluid can be calculated to be 6.2 m^3^ min^−1^ based on formula (3.2). During hydraulic fracturing operation, the upper limit of injection rate of fracturing fluid is set as 5.5 m^3^ min^−1^.

According to formula (3.2), the net pressure in the hydraulic fracture is 6.7 MPa when the injection rate is 5.5 m^3^ min^−1^. The ratio of net pressure to *in situ* stress difference is 0.807, with a corresponding critical thickness ratio of 0.56 (based on figure 15). The theoretical height of a hydraulic fracture in the lower barrier can be calculated to be 8.4 m. Figure 13 shows the array acoustic logging interpretation results before and after fracturing. The results show that the depth of hydraulic fracture in the lower barrier is actually 2173–2183 m after fracturing and the fracture height is 10 m. Compared with the theoretical height (8.4 m), the error is 16%. Similarly, the theoretical height of a hydraulic fracture in the upper barrier is 12.2 m and the detected height of hydraulic fracture is 14 m (depth of 2144–2158 m), with an error of 12.8%.

### 4.2. Well Shen55

**Figure 17.**
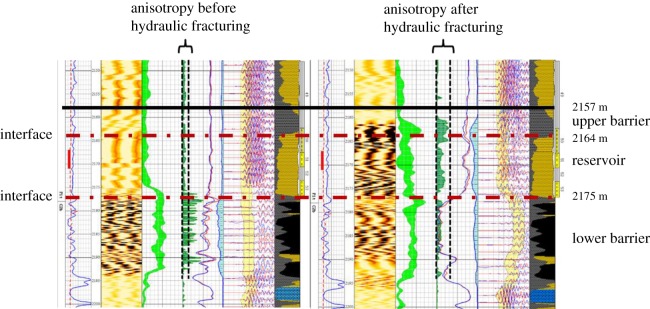
Interpretation results of array acoustic wave logging of Well Shen55.

The production layer is 2164–2175 m deep, and the minimum horizontal principal stress difference between the upper barrier and reservoir is 7.9 MPa (shown in figure 17). The upper barrier is mudstone and lower barrier consists of coal and barrier. The upper barrier is 2157–2164 m deep (with a thickness of 7 m) and the thickness ratio between the upper barrier and reservoir is 0.63. The corresponding net pressure in the hydraulic fracture is 6.68 MPa based on figure 15. The injection rate of fracturing fluid can be calculated to be 5.5 m^3^ min^−1^ based on formula (3.2). During hydraulic fracturing operation, the upper limit of injection rate of fracturing fluid is set as 4.5 m^3^ min^−1^.

According to formula (3.2), the net pressure in the hydraulic fracture is 5.7 MPa when the injection rate is 4.5 m^3^ min^−1^. The ratio of net pressure to *in situ* stress difference is 0.721, with a corresponding critical thickness ratio of 0.39 (based on figure 15). The theoretical height of a hydraulic fracture in the lower barrier can be calculated to be 4.3 m. Figure 14 shows that the depth of hydraulic fracture in the upper barrier is actually 2159–2164 m after fracturing and the fracture height is 5 m. Compared with the theoretical height (4.3 m), the error is 14%. The component of coal in the lower barrier is well bedded, it is difficult to distinguish the height of a hydraulic fracture in lower barrier from figure 15.

The three measurements of fracture height in Well Shen52 and Well Shen55 are at an average error within 14.3% of the theoretical value, which proves the accuracy of the new chart for field application.

## 5. Conclusion

(1) A new chart of the ratio between net pressure and *in situ* stress difference with critical thickness ratio is obtained. Compared with the Gulrajani–Nolte chart, this new chart shows that longitudinal propagation of hydraulic fracture is still finite when the net pressure in fracture reaches or is higher than *in situ* stress difference between reservoir and restraining barrier.

(2) There is a threshold for an injection rate of fracturing fluid to realize hydraulic fracture penetrating interface and propagating in the restraining barrier. When the injection rate is lower than a certain rate, the fracture height remains unchanged with the increase of injection rate. Once the injection rate is increased to exceed this level, the hydraulic fracture can penetrate the interface and propagate in the restraining barrier.

(3) The small ratio of net pressure in the fracture to *in situ* stress difference between reservoir and restraining barrier means a weak capacity of fracture longitudinal propagation. When the ratio is less than 0.56, hydraulic fracture propagation is completely confined to the reservoir.

(4) Hydraulic fracturing parameters in Well Shen52 and Well Shen55 are optimized by using the new chart. The accuracy of the numerical model is indirectly proved by the field case that three measurements of the fracture height are at an average error within 14.3% of the theoretical value.

## 6. Discussion

The new chart is based on geological data of a tight sandstone reservoir in Shenmu block of Changqing Oilfield of China. Interpretation of array acoustic wave logging in Shenmu block shows that the height of hydraulic fracture accords with the new chart, which proves the accuracy of the new chart for field application.

For other reservoirs, whether the new chart is still suitable requires further study. It is recommended to use the method proposed in this paper to set up a specific chart for a particular reservoir.

## Data accessibility

Our data are from the filed case. The numerical parameters are based on geological data of a tight sandstone reservoir in Shenmu block of Changqing Oilfield of China, listed in table 1. Data of Well Shen52 and Well Shen55 are described in detail in field application section of this text.

## Permission to carry out fieldwork

Co-authors Q.T. and W.L. obtained the appropriate permissions to conduct the fieldwork. They are responsible to finish field application section.
